# The NLRP3 Inflammasome Is Dispensable in Methicillin-Resistant *Staphylococcus aureus* Urinary Tract Infection

**DOI:** 10.3390/pathogens13020106

**Published:** 2024-01-25

**Authors:** Santosh Paudel, Rahul Kumar, Kenneth A. Rogers, Yogesh Saini, Sonika Patial, Ritwij Kulkarni

**Affiliations:** 1 Department of Biology, University of Louisiana at Lafayette, Lafayette, LA 70504, USA; psantosh@umich.edu; 2 Department of Population Health and Pathobiology, College of Veterinary Medicine, North Carolina State University, Raleigh, NC 27606, USA; 3 New Iberia Research Center, University of Louisiana at Lafayette, Lafayette, LA 70560, USA; 4 National Institute of Environmental Health Sciences (NIEHS), Research Triangle Park, Durham, NC 27709, USA

**Keywords:** MRSA, UTI, NLRP3, inflammasome

## Abstract

The NLRP3 inflammasome is a cytoplasmic complex that senses molecular patterns from pathogens or damaged cells to trigger an innate immune defense response marked by the production of proinflammatory cytokines IL-1β and IL-18 and an inflammatory death called pyroptosis. The NLRP3 inflammasome is activated in the urinary tract by a variety of infectious and non-infectious insults. In this study, we investigated the role of the NLRP3 inflammasome by comparing the pathophysiology of methicillin-resistant *Staphylococcus aureus* (MRSA) ascending UTI in wild-type (WT) and *Nlrp3*^−/−^ mice. The difference in the bacterial burden detected in the urinary tracts of MRSA-infected WT and *Nlrp3*^−/−^ was not statistically significant at 6, 24, and 72 h post-infection (hpi). The levels of pro-inflammatory cytokines and chemokines as well as the numbers of granulocytes recruited to bladder and kidney tissues at 24 hpi were also similar between *Nlrp3*^−/−^ and WT mice. The histopathological analysis of MRSA-infected bladder and kidney sections from *Nlrp3^−/−^* and WT mice showed similar inflammation. Overall, these results suggest that MRSA-induced urinary NLRP3 activity does not play a role in the pathophysiology of the ascending UTI.

## 1. Introduction

*Staphylococcus aureus* is an atypical cause of asymptomatic bacteriuria and complicated urinary tract infections (UTIs) primarily affecting individuals with indwelling urinary catheters, the elderly, and the hospitalized [1,2,3,4,5,6,7]. *S. aureus* colonization of the urinary tract (UT) is a major clinical concern because of the increased detection of methicillin-resistant *Staphylococcus aureus* (MRSA) in urine specimens in the last two decades [6,8,9] and because it can exacerbate into bloodstream infections (BSIs) secondary to UTI (called uBSI, where u refers to UTI) and their potentially life-threatening sequelae such as sepsis and shock [6,9,10,11]. Previous reports have described specific host–pathogen effectors crucial for the survival and persistence of MRSA in the UT. For example, in the mouse model of catheter-associated UTI (CAUTI), MRSA infection was reported to augment catheter implant-mediated localized pro-inflammatory cytokine response and fibrinogen release in the urinary bladder [12]. We have reported that 2-hour-long, in vitro exposure to human urine increases MRSA virulence and induces expression of metabolic genes necessary for survival in the nutrient-limiting environment of the UT [13] and that in a mouse model of CAUTI, the MRSA mutants in the tricarboxylic acid (TCA) cycle gene *sucD* and the pyruvate oxidation gene *lpdA* are defective in bladder infection and uBSI [14]. In this report, we explored the role of NLRP3 (NOD (nucleotide oligomerization domain) LRR (leucin-rich repeat)-containing receptor, pyrin domain-containing protein 3) inflammasome in MRSA uropathogenesis.

In response to a variety of bacterial molecular patterns, NLRP3 forms a cytoplasmic inflammasome complex with ASC (Apoptosis-associated Speck-like protein containing Caspase activation and recruitment domain) adaptor, and caspase-1, which cleaves pro-IL-1β and pro-IL-18 into active IL-1β and IL-18 and activates pro-inflammatory programmed cell death called pyroptosis via gasdermin cleavage [15]. Previous reports have revealed that uropathogenic *Escherichia coli* (UPEC) virulence factors α-hemolysin [16,17], type 1 fimbriae [18], and Toll/IL-1 receptor-containing (TIR-containing) protein C (TcpC, produced by UPEC strain CFT073) [19] play a crucial role in modulating NLRP3-mediated IL-1β production and pyroptosis in macrophages [16,19,20], neutrophils [21], renal fibroblasts [22], and bladder epithelial cells [18] in a UPEC-strain dependent manner. NLRP3-pyroptosis is an effective defense against acute cystitis as it mediates the exfoliation of bladder epithelium and the subsequent elimination of adherent and intracellular UPEC [17,23]. Indeed, 24 h after experimental induction of ascending UTI with UPEC CFT073, *Nlrp3*^−/−^ mice exhibited significantly higher UT bacterial burden and severe cystitis compared with their wild-type (WT) counterparts, although in UPEC-infected *Nlrp3^−/−^* mice, while IL-1β was processed via a non-canonical, metalloproteinase-7-mediated mechanism [23]. CFT073 factor TcpC has also been reported to interact with NLRP3 and reduce IL-1β production in murine macrophages [19]. Moreover, exfoliation of infected bladder epithelium also exposes underlying cells to uropathogens, suggesting a role for UPEC-α-hemolysin-mediated activation of NLRP3 in priming the UT for chronic infection [24].

Since the role of NLRP3 in the pathogenesis of non-UPEC uropathogens has not been deciphered, we sought to bridge this knowledge gap by comparing MRSA uropathogenesis in WT and *Nlrp3*^−/−^ mice. We hypothesized that NLRP3 inflammasome activity plays a protective role in MRSA acute cystitis. Various *S. aureus* toxins including Panton-Valentine leukocidin (PVL), leukocidin AB (LukAB), and α-hemolysin (Hla) have been reported to trigger the NLRP3 inflammasome in myeloid cells [25,26,27,28]. NLRP3 activity can be protective, detrimental, or dispensable for the host depending on the site of *S. aureus* infection. For example, in the mouse model of S. *aureus* skin and soft tissue infection, NLRP3 activity regulates IL-17 production by γδT cells and is protective for the host [29]; in the mouse model of MRSA pneumonia, NLRP3 suppresses bactericidal activities of macrophages and is detrimental for the host [25]; while in the mouse model of acute central nervous system MRSA infection, NLRP3 is dispensable as it can be replaced with AIM2 (absent in melanoma 2) in the IL-1β processing inflammasome complex [30]. We compared bacterial organ burden, cytokine response, and immune cell recruitment between WT and *Nlrp3*^−/−^ mice infected with MRSA 1369 via the transurethral route.

## 2. Materials and Methods

### 2.1. Bacteria, Mice, and the Reagents

All reagents were purchased from Fisher Scientific unless otherwise specified. Uropathogenic MRSA 1369 [31] was incubated at 37 °C to a mid-log phase (OD_600_ = 0.6). To prepare inoculum for mouse infections, bacteria were washed once in sterile D-PBS (Dulbecco’s phosphate-buffered saline) and adjusted to 10^9^ CFU (colony-forming units)/mL. NLRP3 inhibitor MCC950 was purchased from InvivoGen and resuspended in DMSO vehicle before administration at the desired concentration.

### 2.2. Mouse Models of Ascending UTI and Catheter-Associated UTI (CAUTI)

The mouse experiments were approved by the Institutional Animal Care and Use Committee (IACUC) at the University of Louisiana at Lafayette (2018-8717-011). The *Nlrp3^−/−^* (Stock #021302, The Jackson Laboratory [32]) mouse breeding trio was housed at the biology department mouse facility located at UL Lafayette. To standardize gut microbiota between WT and *Nlrp3^−/−^* mice, we used bedding transfer, where the soiled bedding from the cages housing WT (C57Bl6) and *Nlrp3^−/−^* mice was mixed and distributed equally over a period of three weeks, from 5 to 8 weeks of age [33]. For inducing ascending UTI, we administered via transurethral catheterization 5 × 10^7^ CFU of MRSA 1369 resuspended in 50 µl sterile PBS into the urinary bladders of 8-weeks-old female WT and *Nlrp3^−/−^* mice anesthetized using isoflurane (RXISO-100, Med-Vet International, USA) inhalation [34]. In separate experiments, we induced CAUTI by administering MRSA 1369 via transurethral catheterization immediately after implanting a 5 mm piece of silicone tubing (SIL 025, RenSIL) [12]. Mice were euthanized (CO_2_ inhalation followed by cervical dislocation) at indicated time points. The bladder, kidney, and spleen were dilution plated to determine organ-specific bacterial burden or processed for ELISA, flow cytometry, or histopathology, as described elsewhere.

### 2.3. MCC950 Treatment of the Mouse Model of Ascending UTI

In separate experiments, 4 h after induction of ascending UTI, one group of MRSA 1369-infected WT mice was intraperitoneally injected with 10 mg/kg MCC950 while the control group was injected with DMSO vehicle. Mice were euthanized 24 h post-infection (hpi), and MRSA burden in the bladder, the kidneys, and the spleen was determined by dilution-plating tissue homogenates.

### 2.4. Cytokine Profiling by ELISA

The bladder and kidney homogenates in sterile D-PBS were filtered through a 0.65 µm Ultrafree^®^-MC Centrifugal Filter (UFC30DV0S, Millipore sigma, Burlington, MA, USA), and the total protein concentration was estimated using a Pierce BCA protein assay kit (Thermo Fisher Scientific, Waltham, MA, USA). The levels of cytokines IL-1β, IL-6, IL-10, IL-17A, TNF-α, CXCL1 (KC), CCL2 (MCP-1), CCL3 (MIP1α), CCL5 (RANTES), and IFN-γ in the tissue homogenates were estimated using MILLIPLEX^®^ Mouse Cytokine/Chemokine Magnetic Bead Panel (MCYTOMAG-70K-10C).

### 2.5. Immune Cell Infiltration in the Bladder and the Kidney Tissues

Specific immune cells infiltrating the bladder and the kidneys of WT and *Nlrp3*^−/−^ mice were identified using a panel of fluorescent-labeled antibodies for flow cytometry (Table 1 and [35]). Prior to the antibody treatment, the diced organs were enzymatically digested in RPMI medium containing collagenase IV (8 mg/mL for the bladder and 2 mg/mL for the kidney) and DNase I (1 µL) at room temperature (RT) for 90 min and 250 rpm shaking with frequent pipetting to mix. The cell suspension was passed through a 35 µm filter strainer (Falcon^®^, USA) to remove leftover tissue pieces and washed once in D-PBS (650× *g*, 5 min, RT).

After treatment with RBC lysis buffer (RT, 10 min), the cells were centrifuged. Next, the cell pellets were stained (in tubes protected from light) with 1 µL live/dead marker (Alexa Fluor 430 NHS Ester (Succinimidyl Ester, ThermoFisher) RT, 25 min), 2 µL of Fc block (surface staining, 4 °C, 10 min), and then with an antibody cocktail (2 µL/antibody, RT, 15 min). Between the two staining steps, the cell pellets were washed once in FACS buffer (D-PBS + 2%FBS). After the final staining step, the cells were resuspended in 250 µL fixation buffer (4 °C, 20 min), washed once in FACS buffer, and resuspended in FACS buffer for use in flow cytometry. The data were analyzed with FlowJO^TM^ version 10 (BD Life Sciences, Franklin Lakes, NJ, USA). After gating on CD45^+^ cells, we detected monocytes (MHCII^−^ CD11b^+^ Ly6G^−^), neutrophils (MHCII^−^ CD11b^+^ Ly6G^+^), eosinophils (MHCII^−^ CD11b^+^ SiglecF^+^ Ly6G^−^), and mast cells (CD117^+^) using the gating strategy described in Figure S1.

### 2.6. Histopathological Examination of the Bladder and Kidney

WT and *Nlrp*3^−/−^ mouse bladders and kidneys (from MRSA-infected and control mice) were preserved in 10% formalin, embedded in paraffin, sectioned, and stained with hematoxylin and eosin. The severity and extent of inflammation in each section were scored in a blinded manner by a veterinary pathologist using a published semiquantitative scoring scheme [36]. For bladder sections, widespread inflammation, thrombosed vessels, and marked submucosal edema were assigned a score of 3; mixed inflammation in mucosa and submucosa and around vessels, a score of 2; scattered neutrophils in the submucosa and migrating through the mucosa, a score of 1; while normal sections were assigned a score of 0. For kidney sections, many neutrophils in the pelvic lumen and within the tissue were assigned a score of 3; clustered neutrophils in the pelvic lumen and inflammation within the epithelium and surrounding stroma, a score of 2; scattered neutrophils migrating through the pelvic epithelium, a score of 1; while normal sections were assigned a score of 0.

### 2.7. Statistical Analysis

The data were analyzed using GraphPad Prism 10. Data from multiple biological replicates with two or more technical replicates for each experiment were pooled together. Error bars in the figures represent standard deviation. Organ burden, cytokine amount, number of infiltrating immune cells, and histological scores between the WT and the *Nlrp*3^−/−^ mice were compared using the Mann–Whitney U statistic. The numbers of WT and *Nlrp3*^−/−^ spleen homogenates with MRSA CFUs and the numbers of WT and *Nlrp3*^−/−^ bladders from which we could recover catheter implants were compared using Fisher’s exact test. Data were considered statistically significant if *p* ≤ 0.05.

## 3. Results

### 3.1. The Pathophysiology of MRSA UTI in WT and Nlrp3^−/−^ Mice

**Figure 1:**
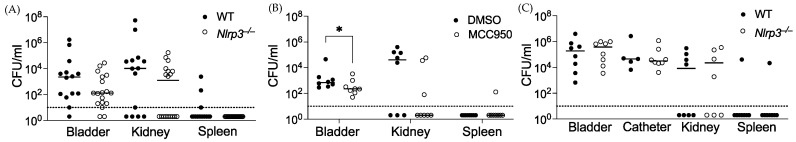
The effects of NLRP3 ablation on MRSA CFUs in the murine urinary tracts. Female WT (control) and *Nlrp3*^−/−^ mice were inoculated transurethrally with 5 × 10^7^ CFU of uropathogenic MRSA strain, MRSA 1369. Mice were euthanized, and the bacterial burden in the bladder, the kidneys, and the spleen were determined at 24 hpi (**A**). In separate experiments, WT C57BL6 mice infected with MRSA1369 were injected 4hpi intraperitoneally with either 10 mg/kg MCC950 or DMSO vehicle. MRSA CFUs in the bladder, the kidneys, and the spleen were determined at 24 hpi (**B**). At 24 hpi, MRSA 1369 organ burden in the UT and catheter insert was also determined in the WT and *Nlrp3*^−/−^ mouse model of CAUTI (**C**). Scatter plots show CFU counts from individual mice (*n* = 6 to 18/group) with the median as the measure of central tendency; the dotted lines show the limit of detection. The data from 3 (**A**) or 2 (**B**,**C**) independent experiments are shown. Statistical significance was determined with a Mann–Whitney U test. For all figures, *p* ≤ 0.05 was considered significant and indicated by *.

To examine the effects of the NLRP3 inflammasome on the pathophysiology of acute UTI, we inoculated MRSA 1369 in C57BL6 WT and *Nlrp3^−/−^* mice via transurethral catheterization and enumerated CFU burden. At 24 hpi, compared to their WT counterparts, MRSA-infected *Nlrp*3^−/−^ mice showed a statistically insignificant reduction in the median bladder (WT = 2250 CFU/mL, *Nlrp*3^−/−^ = 130 CFU/mL; *p* = 0.22) and median kidney CFUs (WT = 10,250 CFU/mL, *Nlrp*3^−/− =^ 1251 CFU/mL; *p* = 0.12); we detected MRSA CFUs in the spleens of 3/14 WT and 0/18 *Nlrp3*^−/−^ mice (Figure 1A). MRSA CFUs in the bladder, kidney, and spleen homogenates from WT and *Nlrp3*^−/−^ mice were also not significantly different at either 6 hpi (Figure S2A) or 72 hpi (Figure S2B).

Interestingly, when NLRP3 was ablated by treating MRSA 1369-infected WT mice at 4 hpi with NLRP3 inhibitor MCC950, the median bladder bacterial burden in MCC950-treated mice was significantly reduced compared with that in vehicle (DMSO)-treated control mice at 24 hpi (MCC950 = 690 CFU/mL, DMSO = 225 CFU/mL; *p* = 0.049, Figure 1B). However, MCC950 treatment did not significantly affect either the median kidney CFUs (MCC950 < LOD, DMSO = 225 CFU/mL, *p* = 0.2) or the number of mice with kidney bacterial burden (MCC950 = 3/8, DMSO = 5/8, *p* = 0.6 by Fisher’s exact test) (Figure 1B). We confirmed that MCC950 does not directly affect MRSA 1369 survival based on the similar CFUs observed in MRSA 1369 cultivated in vitro for 24 h in the presence of either MCC950 or DMSO vehicle (Figure S3).

Since MRSA UTI is predominantly associated with indwelling urinary catheter use [6], we modeled CAUTI by placing, via the urethra, a small piece of silicone tubing into the bladder of WT and *Nlrp3^−/−^* at the time of infection [14]. At 24 hpi, the median MRSA CFUs in the bladder (WT = 192,000 CFU/mL, *Nlrp*3^−/−^ = 385,000 CFU/mL; *p* = 0.72), catheter implant (WT = 46,000 CFU/mL, *Nlrp*3^−/−^ = 31,500 CFU/mL; *p* = 0.52), and kidney (WT = 8501 CFU/mL, *Nlrp*3^−/−^ = 22,950 CFU/mL; *p* = 0.48) tissues were not significantly different between WT and *Nlrp3^−/−^* mice; the median CFU in the spleen was below the limit of detection in both WT and *Nlrp3^−/−^* (Figure 1C). We observed similar median MRSA CFUs in the bladder, catheter, kidney, and spleen of WT and *Nlrp3*^−/−^ CAUTI mice at 72 hpi (Figure S4A) and 7 days pi (Figure S4B). We recovered catheter implants from 5/8 WT and 8/8 *Nlrp3*^−/−^ mice at 24 hpi and 6/8 WT and 4/8 *Nlrp3*^−/−^ mice at 72 hpi. 

Overall, these results indicated that NLRP3 is dispensable for MRSA ascending UTI and MRSA CAUTI.

### 3.2. Acute MRSA 1369 UTI Induces Similar Cytokine Production and Immune Cell Recruitment to the UT of WT and Nlrp3^−/−^ Mice

Next, we determined the levels of cytokines (multiplex ELISA) in and immune cell recruitment (flow cytometry) to the bladders and kidneys from the WT and *Nlrp3*^−/−^ mice at 24 hpi, when MRSA 1369 is reported to induce significant localized inflammation in murine bladders [12]. 

**Figure 2:**
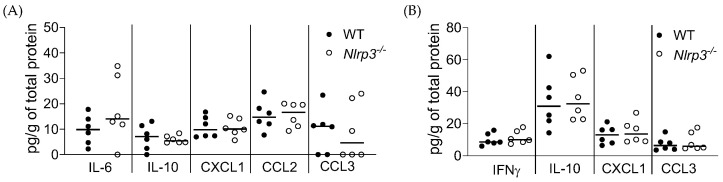
Cytokine profiling of MRSA-infected WT and *Nlrp3^−^*^/−^ mouse urinary tracts. MRSA 1369-infected female WT (control) and *Nlrp3*^−/−^ mice were euthanized at 24 hpi. The levels of specific cytokines in MRSA-infected bladder (**A**) and kidney (**B**) homogenates from WT or *Nlrp3*^−/−^ mice were quantified with Multiplex-ELISA. The combined data from two or more independent experiments are shown.

Figure 2 shows that the IL-6, IL-10, CXCL1, CCL2, and CCL3 levels in WT and *Nlrp3*^−/−^ mouse bladders (Figure 2A) and the IFNγ, IL-10, CXCL1, and CCL3 levels in WT and *Nlrp3*^−/−^ mouse kidneys (Figure 2B) were above the limit of detection but not significantly different. The remaining cytokine levels were below the level of detection.

**Figure 3:**
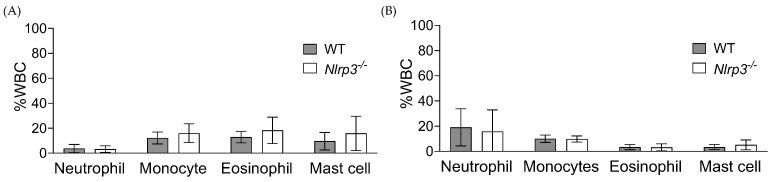
Immune cell infiltration to MRSA-infected WT and *Nlrp3^−^*^/*−*^ mouse urinary tracts. The bladder (**A**) and kidney (**B**) homogenates from WT (*n*= 9) and *Nlrp3*^−/−^ (*n* = 7) mice infected with MRSA 1369 for 24 h were analyzed with flow cytometry. Specific lymphocyte types are shown as the percentage of CD45^+^ lymphocytes. The combined data from two or more independent experiments are presented as mean *±* standard deviation.

Using the fluorescent antibodies to stain specific cell surface markers (Table 1) and the gating strategy (Figure S1), we differentiated between different immune cells. Among the CD45^+^ cells, MHCII^−^/CD11b^+^/Ly6G^+^-neutrophils, MHCII^−^/CD11b^+^/Ly6G^−^-monocytes, MHCII^−^/CD11b^+^/SiglecF^+^/Ly6G^−^-monocytes and CD117^+^-mast cells were dominant. However, compared to their WT counterparts, neither *Nlrp3*^−/−^ bladders nor *Nlrp3*^−/−^ kidneys showed significantly different recruitment of neutrophils, monocytes, eosinophils, or mast cells (Figure 3A,B). 

### 3.3. Histopathological Examination of WT and Nlrp3^−/−^ Bladder and Kidney Sections

**Figure 4:**
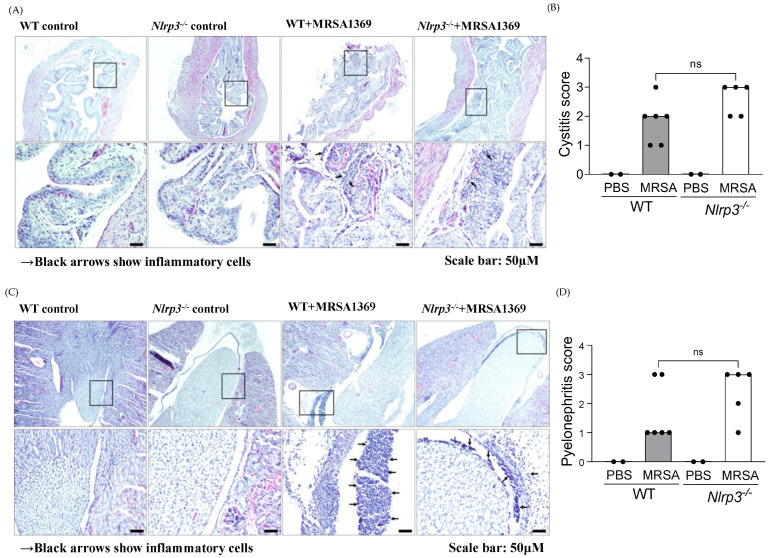
Histopathological examination of MRSA-infected WT and *Nlrp3^−^*^/*−*^ bladder and kidney sections. Bladder (**A**) and kidney (**C**) sections from control (PBS) and MRSA-infected WT and *Nlrp3^−^*^/*−*^ mice were stained with hematoxylin–eosin to visualize inflammatory cells (shown with an arrow). The tissue sections were scored in a blinded manner using the specific criteria listed in the material and methods. The inflammation scores for individual bladder (**B**) and kidney (**D**) samples are presented as a scatter plot with the median as the measure of central tendency. The inflammation score (**B**,**D**) data are from tissue sections from two independent experiments. Statistical significance was determined with the Mann–Whitney U test.

In comparison with the PBS-inoculated controls, MRSA-infected WT as well as *Nlrp3*^−/−^ mice showed a significantly higher presence of inflammatory cells in bladders (Figure 4A) and kidneys (Figure 4C). We observed that MRSA infection significantly increased the median inflammation scores in the bladder (Figure 4B) and kidney (Figure 4D) of both WT and Nlrp3^−/−^ mice compared with PBS-inoculated controls. However, the severity of inflammation in either MRSA-infected bladder or MRSA-infected kidney tissues between WT and Nlrp3^−/−^ mice was not significantly different.

## 4. Discussion

Various reports have established a crucial role for the NLRP3 inflammasome in acute and chronic cystitis caused by uropathogenic *E. coli* (UPEC) [16,17,18,19,20,21,22,23,24]. Whether the NLRP3 inflammasome is important in MRSA-UTI, however, has not been deciphered. In this report, we sought to bridge this knowledge gap by comparing MRSA UTI between C57BL6 WT and *Nlrp3*^−/−^ mice carrying a targeted mutation in the *Nlrp3* gene [32]. Our central hypothesis was that NLRP3 inflammasome activity plays a protective role in MRSA acute cystitis. The in vitro exposure to human urine induces the expression of MRSA-1369 α-hemolysin [13], which is a known activator of NLRP3 in myeloid cells [26]. As a model organism, we used uropathogenic MRSA 1369, which has been previously used in UTI research [12,13,14]. We examined MRSA organ burden in the WT and *Nlrp3*^−/−^ mice either in the absence or the presence of the catheter implant. Examining MRSA infection in the mouse model of CAUTI is clinically relevant because the use of an indwelling urinary catheter is a known risk factor for MRSA-UTI [7]. The choice of time points (6, 24, and 72 hpi for ascending UTI without catheter insert and 72 hpi and 7 days pi for CAUTI) for organ burden comparison was driven by previous reports that WT mice cleared MRSA infection around 96 hpi in the absence of a catheter implant [37], while MRSA CFUs were detected until 14 days pi in a mouse model of CAUTI [12].

We observed a consistent but statistically insignificant reduction in the CFUs recovered from the bladder and kidneys of *Nlrp3*^−/−^ mice compared to the WT at 24 and 72 h after the induction of ascending UTI without catheter implants. MRSA burden was detected in fewer *Nlrp3*^−/−^ mouse kidneys compared to the WT, although this difference was also not statistically significant. In contrast to these results, in the mouse model of CAUTI, the median MRSA CFUs recovered from the bladder and the kidneys, the overall spread of the data around the median, and the number of mice with detectable CFU burden were similar between the WT and *Nlrp3*^−/−^ backgrounds at 24 and 72 hpi. Even at 7 days pi, the CFU difference between WT and *Nlrp3*^−/−^ MRSA burden was not statistically different. The administration of NLRP3 inhibitor MCC950, 4 h after the induction of ascending UTI without a catheter, resulted in a 3-fold, statistically significant reduction in bladder CFUs at 24 hpi and a corresponding reduction in kidney CFUs that was statistically non-significant. MCC950 is a small molecular inhibitor that selectively inhibits the formation of NLRP3 inflammasome complex by binding the NLRP3 NACHT domain and blocking ATP hydrolysis [38,39].

In addition, the immune profiling of mice revealed that NLRP3 activity does not affect either cytokine production or immune cell infiltration in the MRSA-infected UT. The histopathological examination of bladder and kidney sections showed that MRSA 1369-induced inflammation was not significantly different between WT and *Nlrp3*^−/−^ mice. It has been previously reported that MRSA 1369 infection exacerbates catheterization-induced localized inflammation at 24 hpi [12]; the bladder and the kidneys from the WT mice infected without a catheter implant with MRSA strain SA116 also showed higher levels of pro-inflammatory cytokines, IL-1β, IL-6, and TNFα at 24 hpi [37]. In contrast, we observed that MRSA 1369 mediates only a modest increase in the levels of various pro-inflammatory cytokines and chemokines in the non-catheterized murine UT at 24 hpi. This discrepancy may be attributed either to the catheter implant or to the potential differences between SA116 and MRSA 1369.

## 5. Conclusions

In summary, the activation of the NLRP3 inflammasome appears to be dispensable during MRSA acute UTI. Since we examined infection parameters in WT and *Nlrp3*^−/−^ mice up to 72 h after the induction of ascending UTI without a catheter, future experiments focused on determining (i) whether MRSA-induced NLRP3 activity induces exfoliation of the uroepithelium, in turn promoting chronic MRSA-UTI, similar to what is reported in UPEC-UTI, and (ii) whether urinary NLRP3 activity shapes the pathophysiology of life-threatening exacerbations such as uBSI following MRSA CAUTI are warranted.

## Figures and Tables

**Table 1:** List of antibodies used for flow cytometry.

Antibodies	Conjugate	Clone
CD64	BV 786	X54-5/7.1
SiglecF	BV711	M290
CD3	BV650	145-2C11
CD45	BV605	30-F11
Live/Dead	Alexa fluor 430	
Fc epsilon RI	Pac Blue	MAR1
Ly6C	PE-Cy7	HK1.4
CD11c	PerCp-Cy5.5	N418
CD103	PE-CF594	M290
c-kit (CD117)	PE	2B8
CD11b	FITC	M1/70
MHC-II (I-A/I-E)	APC-Fire750	M5/114.15.2
F4/80	Alexa Fluor 700	CI:A3-1

## Data Availability

The data presented in this study are available in the body of this article or the online Supplementary Materials.

## Supplementary Materials

The following supporting information can be downloaded at: https://www.mdpi.com/article/10.3390/pathogens13020106/s1, Figure S1: The gating strategy for flow cytometry; Figure S2: MRSA 1369 organ burden in the WT and *Nlrp3^−^/^−^* mouse model of ascending UTI (without a catheter insert) at 6 and 72 hpi; Figure S3: MCC950 does not affect the in vitro growth of MRSA 1369 in brain heart infusion (BHI) broth; Figure S4: MRSA 1369 organ burden in the WT and *Nlrp3^−^/^−^* mouse model of CAUTI at 72 hpi (A) and 7 days pi (B).
