# Supplementary material for: The NLRP3 Inflammasome Is Dispensable in Methicillin-Resistant Staphylococcus aureus Urinary Tract Infection
Source: Pathogens. 2024 Jan 25;13(2):106. doi: 10.3390/pathogens13020106 (PMC10893321; doi:10.3390/pathogens13020106)

Fig. S1. The gating strategy for flow cytometry.

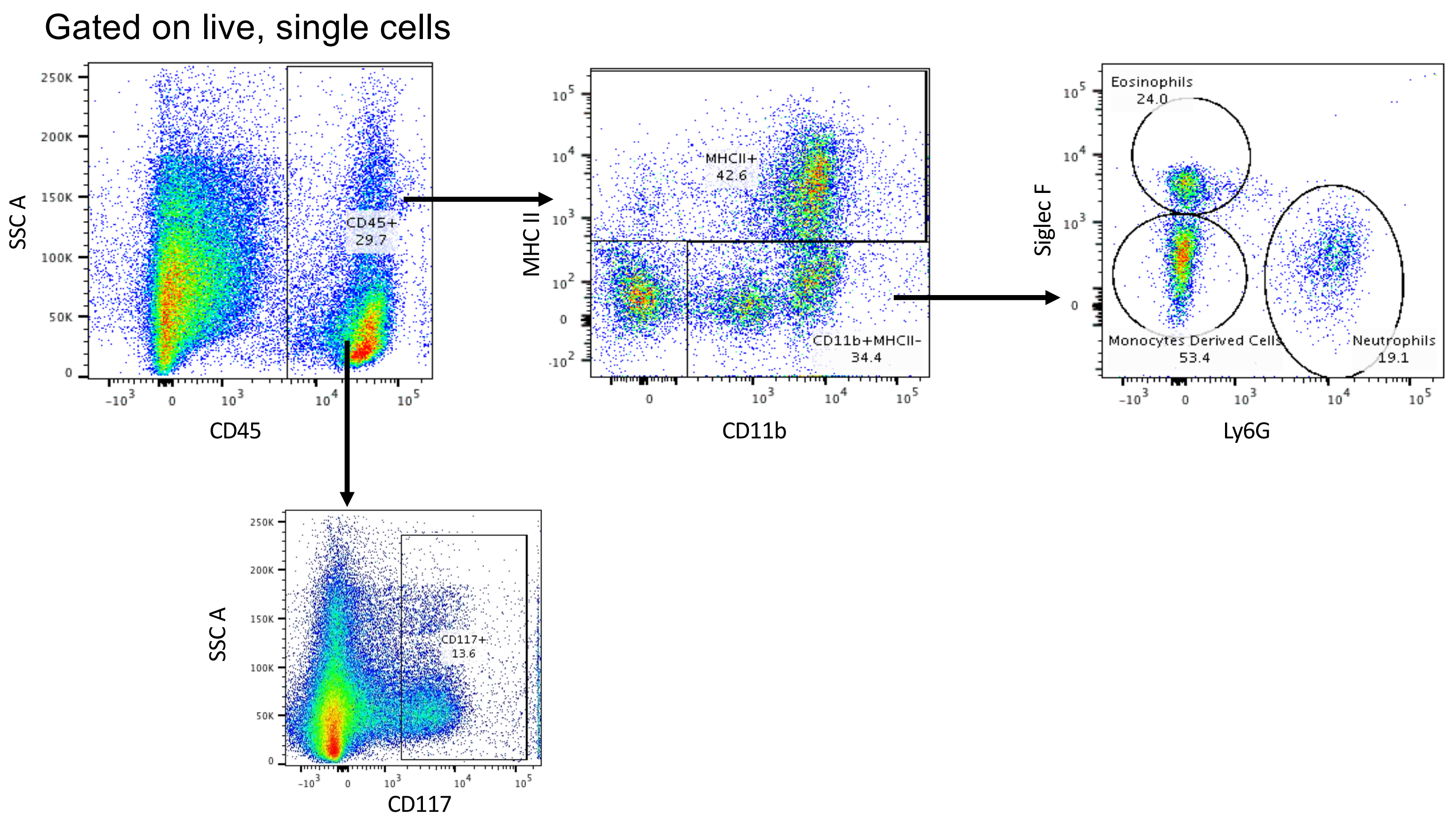

Fig. S2. MRSA 1369 organ burden in the WT and *Nlrp3*$^{-/-}$ mouse model of ascending UTI (without a catheter insert) at 6 and 72 hpi

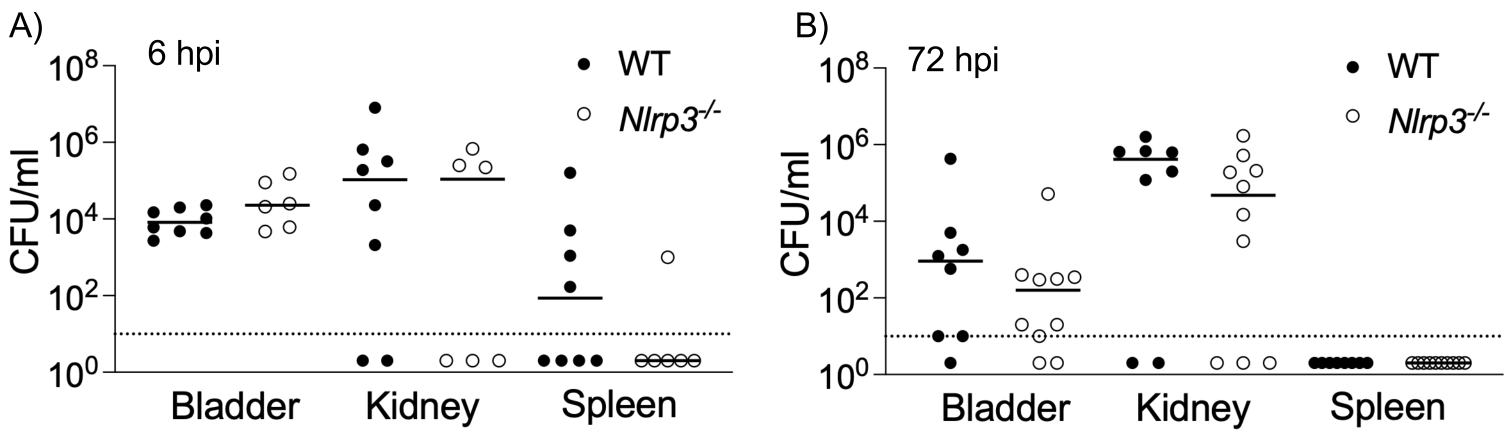

Fig. S3. MCC950 does not affect the *in vitro* growth of MRSA 1369 in brain heart infusion (BHI) broth

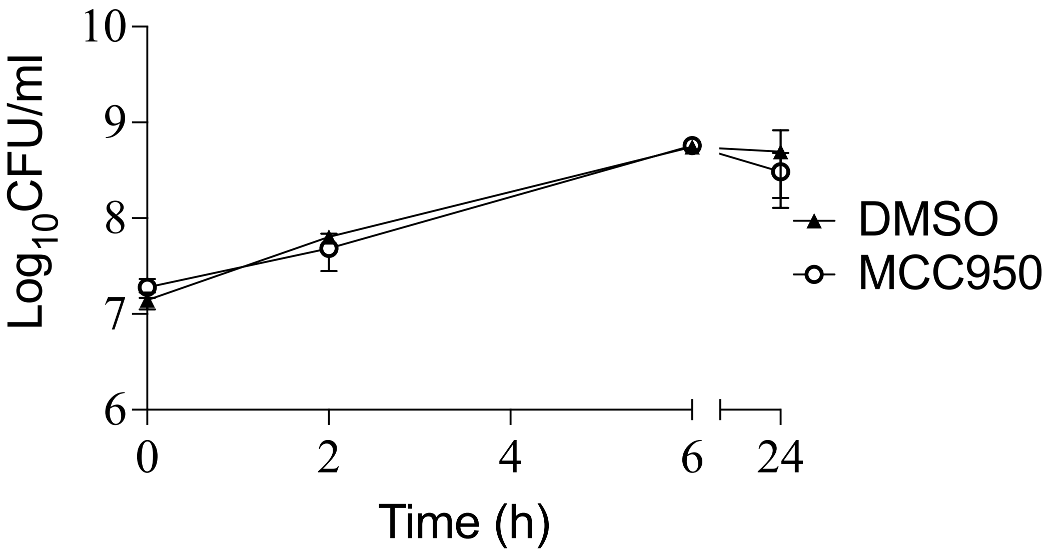

Fig. S4. MRSA 1369 organ burden in the WT and *Nlrp3-/-* mouse model of CAUTI at 72 hpi and 7 days pi

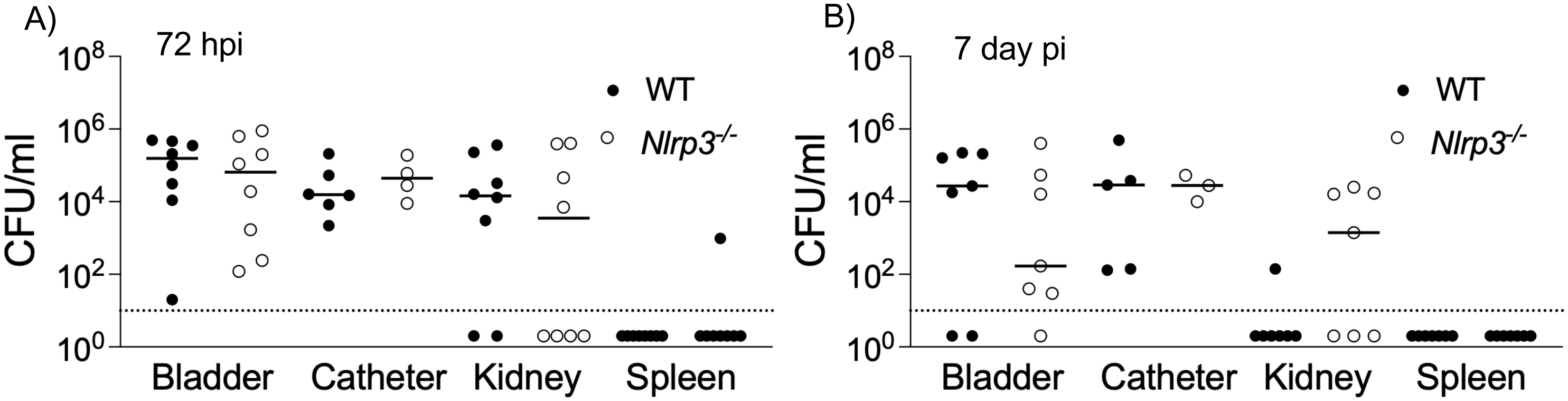

Supplement: Supplementary file 1 [file pathogens-13-00106-s001.zip › pathogens-2750825-supplementary.pdf]
